# Two-step functional screen on multiple proteinaceous substrates reveals temperature-robust proteases with a broad-substrate range

**DOI:** 10.1007/s00253-021-11235-9

**Published:** 2021-03-26

**Authors:** Antonio García-Moyano, Yuleima Diaz, José Navarro, David Almendral, Pål Puntervoll, Manuel Ferrer, Gro Elin Kjæreng Bjerga

**Affiliations:** 1 NORCE Norwegian Research Centre, Thormøhlens gate 55, 5006 Bergen, Norway; 2 Institute of Catalysis, Consejo Superior de Investigaciones Científicas, 28049 Madrid, Spain

**Keywords:** Functional metagenomics, Agar screen, Protease M10/M12, Zein

## Abstract

**Abstract:**

To support the bio-based industry in development of environment-friendly processes and products, an optimal toolbox of biocatalysts is key. Although functional screen of (meta)genomic libraries may potentially contribute to identifying new enzymes, the discovery of new enzymes meeting industry compliance demands is still challenging. This is particularly noticeable in the case of proteases, for which the reports of metagenome-derived proteases with industrial applicability are surprisingly limited. Indeed, proteolytic clones have been typically assessed by its sole activity on casein or skim milk and limited to mild screening conditions. Here, we demonstrate the use of six industry-relevant animal and plant by-products, namely bone, feather, blood meals, gelatin, gluten, and zein, as complementary substrates in functional screens and show the utility of temperature as a screening parameter to potentially discover new broad-substrate range and robust proteases for the biorefinery industry. By targeting 340,000 clones from two libraries of pooled isolates of mesophilic and thermophilic marine bacteria and two libraries of microbial communities inhabiting marine environments, we identified proteases in four of eleven selected clones that showed activity against all substrates herein tested after prolonged incubation at 55 °C. Following sequencing, in silico analysis and recombinant expression in *Escherichia coli*, one functional protease, 58% identical at sequence level to previously reported homologs, was found to readily hydrolyze highly insoluble zein at temperatures up to 50 °C and pH 9–11. It is derived from a bacterial group whose ability to degrade zein was unknown. This study reports a two-step screen resulting in identification of a new marine metagenome-derived protease with zein-hydrolytic properties at common biomass processing temperatures that could be useful for the modern biorefinery industry.

**Key points:**

*• A two-step multi-substrate strategy for discovery of robust proteases.*

*• Feasible approach for shortening enzyme optimization to industrial demands.*

*• A new temperature-tolerant protease efficiently hydrolyzes insoluble zein.*

**Supplementary Information:**

The online version contains supplementary material available at 10.1007/s00253-021-11235-9.

## Introduction

The market for novel enzymes and their uses in more environmentally friendly industrial and speciality applications is growing. The global enzyme market is dominated by food and feed enzymes (Chapman et al. 2018), including protease/peptidase, amylase, and lipase/esterase enzymes. The protease market was valued at 1700 million US$ in 2018 and is projected to reach 2630 million US$ by the end of 2025, growing at a compound annual growth rate (CAGR) of 5.6% (Global Protease Market Report 2019). Recombinant proteases have been applied in industrial biotechnology for decades, such as in the dairy industry, where recombinant chymosin is used in cheese making (Mistry 2006). Alongside, market growth has been driven by the detergent industry, among others, where proteases have been added as ingredients in «greener» household cleaning products and soaps (Maurer 2004). However, the livestock industry is projected to be one of the drivers of future demands for the protease market, particularly in the production of new feed ingredients, such as protein hydrolysates from animal co- or by-products (e.g., bone, feathers, skin, blood from meat, poultry, and fish filet processing) and vegetable ingredients from corn, wheat, etc. (Philipps-Wiemann 2018). The main protein sources in these by-products are collagen, gelatin, keratin, serum albumins and globulins, zein, glutenin, and gliadin, respectively. Some of these sources are known to be recalcitrant and poorly soluble, and the lack of suitable enzymes have limited their application as functional food or feed ingredients (Kong et al. 2007; Anderson and Lamsal 2011; Pal and Suresh 2016; Li 2019). Zein, for example, contains more than 50% hydrophobic amino acids, likely creating extensive intermolecular interactions that act as barriers for enzymes to access and hydrolyze the peptide bonds (Anderson and Lamsal 2011). Enzymatic hydrolysis has been shown not only to increase the aqueous solubility of zein (Zhu et al. 2008) and increase its antioxidant and anti-inflammatory activity on endothelial cells and cytotoxic effects on cancer cells (Kong and Xiong 2006; Tang et al. 2010; Jin et al. 2016; Díaz-Gómez et al. 2018; Liang et al. 2018) but also to improve its properties as biological active carrier for encapsulation of bioactive components (Kasaai 2018). To support future demands, advancements of technologies for enzyme discovery are key to accelerate the use of alternative protein sources in feed and food.

The advancement of high-throughput sequencing of metagenomic DNA at gradually declining costs has expanded the knowledge of microbial diversity and made this diversity accessible for studies of novel biocatalysts (Ngara and Zhang 2018). Since most microbes in an environmental sample are expected to be non-cultivable under standard conditions in the lab, clone libraries of environmental DNA are transferred to surrogate bacterial hosts, such as *Escherichia coli*, and functionally characterized through heterologous expression or sequencing. Functional characterization by screens is a common way to discover new enzymes. Such screens include detection of easy-readable phenotypes from single clones in a reporter assay. Since the first report of enzyme discovery from metagenomes in 1995 (Healy et al. 1995), the development of new ways to screen has been slow (Lee and Lee 2013; Ngara and Zhang 2018) and the fraction of this biodiversity that has been subjected to studies on enzyme discovery remains low (Ferrer et al. 2016). Some of the challenges for the discovery of new industrial enzymes by functional screening of metagenomic libraries include the lack of suitable screens with industry-relevant substrates and industry-mimicking conditions (Morris and Marchesi 2015; Ferrer et al. 2016). The classical protease-positive phenotype sought after in functional screening appears by incubating a clone library on skim milk agar (SMA) or casein-supplemented solid medium and detecting clearing zones around the clone that contains the capacity to hydrolyze the substrate. Although it was previously reported that the SMA screen is not sufficient as a definitive screen to identify proteases (Jones et al. 2007), the number of false positives is typically not exceeding the number of true positive clones. Moreover, the absence of reported false negatives (actual proteolytic clones that respond as negative against milk proteins) and the description of alternatives to overcome the bias, e.g., casein or lactose-free skim milk (Morris et al. 2012), make skim milk and casein remain valid substrates for the initial identification of proteolytic metagenomic clones.

In spite of an average frequency of about 10 serine proteases per prokaryotic genome (Tripathi and Sowdhamini 2008) and a relative higher mean incidence rate of proteolytic clones in environmental DNA libraries when performing functional screens, compared to other enzyme classes (e.g., lipases/esterases or glycosidases) (Ferrer et al. 2016), the reports of metagenome-derived proteases are still limited (Kennedy et al. 2011). In comparison, both lipases/esterase and glycosidases have lower estimated incidence rates, but the amount of reports in the literature are several fold more frequent (Ferrer et al. 2016). In fact, the outcome from functional screens for proteases in metagenomic libraries over the past 15 years is limited to 23 active proteases (Table 1) for which 50% of them belong to the well-known S8 family (subtilisins). The reported proteases are almost exclusively identified by sole screening on casein or skim milk agar and in limited experimental conditions (typically incubation at 30–37 °C sometimes prolonged to several days). Their utility against other relevant substrates (substrate range) and their biotechnological potential remains ambiguous (Morris and Marchesi 2015). Although several factors may have contributed to the shortage of protease reports, the choice of substrate creates a bias in the selection of the activities of interest. The introduction of a two-step strategy with an initial selection with a general substrate followed by a more specific one is a recommended approach (Ferrer et al. 2016). In fact, the development of multi-substrate approaches applied to functional screening of microbial metagenomes has resulted in highly efficient discovery rates for other enzyme classes (Schückel et al. 2016; Maruthamuthu et al. 2016). However, such approach has not yet been explored for proteases in a systematic manner.

**Table 1:** Chronological overview of previous agar-based functional metagenomics campaigns in which proteases have been targeted

Reference	Year	Metagenomic source	Screened clones	Protease positive clones	Number proteases characterized	MEROPS family	Substrate for functional assay	Assay temperature (and time)	UniProt ID (characterized proteases)
(Lee et al. 2007)	2007	Coastal marine mud	30,000	1	1	M12A	Skim milk	N/A (N/A)	A1E2A7
(Waschkowitz et al. 2009)	2009	Garden soil, coastal marine, and river sediment	80,000	4	2	M4	Skim milk	30 °C (N/A)	B8PZW2, B8PZX3
(Neveu et al. 2011)	2011	Dessert surface sand	47,000	17	2	S8A	Skim milk	37 °C (24–48 h)	F4ZE69, F4ZE70
(Pushpam et al. 2011)	2011	Goat skin	70,000	1	1	S8A	Skim milk	37 °C (48–72 h)	E2GMY5
(Zhang et al. 2011)	2011	Coastal marine sediment	5000	6	1	S8A	Casein	30 °C (N/A)	B3GVW5
(Niehaus et al. 2011)	2011	Cement plant	336,255	8	3	S8A (2), S1	Skim milk	37 °C (24 h, moved to RT for 4 weeks)	D7GN97, D7GN98, D7GN99
(Biver et al. 2013)	2013	Forest soil	35,000	1	1	S8A	AZCL-casein	37 °C (48 h, moved to RT for 3 weeks)	T1RQV7
(Morris and Marchesi 2015)	2015	Activated sludge dairy plant	28,032	1	2	M48, M4	Skim milk	37 °C (72 h)	K8F7M2, K8ES21
(Iqbal et al. 2014)	2014	Desert soil	700,000	3	3	M20F, M14A, S8A	Antibiosis*	30 °C (24 h, moved to RT for 5 days)	A0A059U0C8, A0A059TX89, A0A059U0E3
(Devi et al. 2016)	2016	Activate sludge tannery	10,000	1	1	S8A	Skim milk	37 °C (48–72 h)	X5F9W5
(Apolinar–Hernández et al. 2016)	2016	Tropical underground water	21,000	23	2	S8A	Skim milk	N/A	A0A1J0F5F1, A0A1J0F5F2
(Gong et al. 2017)	2017	Oil-polluted mudflat	35,000	8	1	M48	Casein	N/A	A0A0X9HI21
(Pessoa et al. 2017)	2017	Mangrove sediment	Unknown	Unknown	1	M42	Skim milk	N/A	Not published
(Jiang et al. 2017)	2017	Polluted agricultural soil	30,000	1	1	S1C	Skim milk	37 °C (24–72 h)	A0A0E3M772
(Sun et al. 2020)	2020	Marine sediment	20,000	4	1	S8A	Skim milk	37 °C (1–7 days)	MG930767

*The library was screened for clones with antibacterial activity in a top agar overlay assay against *Bacillus subtilis*

*N/A* not available, *RT* room temperature

In this study, we aimed at exploiting a new strategy to discover new temperature-robust proteases with broad-substrate range that may be better fit for industrial applications. In order to achieve that goal, we have introduced a two-step screen procedure: a first screen on the general skim-milk substrate followed by a more targeted parallel screen in an expanded repertoire of industry-relevant substrates and conditions.

## Material and methods

### Metagenomic libraries and fosmid clone origin

The collection of proteolytic clones originated from 2 mix-genomic and 2 metagenomic libraries, containing unique marine environmental DNA, previously constructed in the pCCFOS1 large insert fosmid vector using the CopyControl fosmid library production kit (Epicentre, USA). They include 2 libraries made from, respectively, 251 thermophilic marine bacterial strains (TB library) and 194 mesophilic marine bacterial strains (MB library) that were isolated from enrichment cultures from deep sea hydrothermal vents and used to prepare “mixed genome” libraries (Leis et al. 2015). Also, two libraries from microbial communities inhabiting the Milazzo polluted harbor (MH libraries) were included, one origining from seawater and one from sediment (Martínez-Martínez et al. 2018). *E. coli* EPI300-T1^R^ (Epicentre, USA) was used as host for all metagenomic libraries.

### Multi-substrate functional agar-based screens

Fosmid clone libraries were first screened on 22.5 × 22.5 cm Petri plates with SMA (1% (w/v) tryptone, 0.5% (w/v) yeast extract, and 0.5% (w/v) NaCl, 1.5% (w/v) agar, and 1% (w/v) skim milk (Sigma-Aldrich, USA)) supplemented with L-arabinose (0.1% (w/v)) to induce a high fosmid copy number and 15 μg/ml chloramphenicol for fosmid selection. Plates were incubated at 37 °C overnight for microbial growth, and for 3 more days at room temperature to develop putative proteolytic halos. The fosmid clones selected as “proteolytic” based on this classical skim-milk hydrolysis assay were used as a starting point in the multi-substrate screen (Table 2).

**Table 2:** Summary of the fosmid clone libraries and protease identity. Proteolytic fosmid ID representing individual positive clones is based on a two-letter code, firstly describing the environmental or microbial source (TB, MB, and MH, referring to thermophilic bacteria, mesophilic bacteria, and Milazzo harbor, in this order), followed by an arbitrary number representing the appearance of clones found to be active in each source. Protease candidates that were subjected to downstream analysis were given an arbitrary identification number (C1 to C6)

Library source (number of screened clones)	Proteolytic fosmid ID	GenBank accession number	Candidate protease ID	Query location	% identity	RAST functional annotation	MEROPS classification
Thermophilic marine bacterial strains (80,000 clones)	TB1	SAMN14533298	C1	[10943–9867]	99.44	Glutamyl aminopeptidase	M42
			C2	[21222–19807]	83.58	Dipeptidase	M20A
			C3	[28383–26737]	98.45	Thermolysin	M04
	TB2		C1	[5335–4259]	99.44	Glutamyl aminopeptidase	M42
			C2	[15616–14204]	83.58	Dipeptidase	M20A
			C3	[22679–21033]	98.45	Thermolysin	M04
			C4	[40479–39691]	99.24	Dipeptidyl aminopeptidase	S09B
	TB3		C2	[6587–5175]	83.58	Dipeptidase	M20A
			C3	[13743–12097]	98.45	Thermolysin	M04
			C4	[31543–30755]	99.24	Dipeptidyl aminopeptidase	S09B
	TB4	-	-	-	-	-	-
Mesophilic marine bacterial strains (20,000 clones)	MB1	-	-	-	-	-	-
	MB2	-	-	-	-	-	-
	MB3	-	-	-	-	-	-
Milazzo polluted harbor sediment (40,000 clones)	MH1	SAMN14533297	-	[8041–8778]	73.36	Rhomboid protease	S54
			C5	[18847–17378]	78.21	HtrA protease	S01C
			C6	[29254–30123]	58.02	Metallopeptidase	M10A
Milazzo polluted harbor seawater (200,000 clones)	MH2	-	-	-	-	-	-
	MH3	-	-	-	-	-	-
	MH4	-	-	-	-	-	-

This collection of proteolytic clones was propagated and maintained in LB medium (1% (w/v) tryptone, 0.5% (w/v) yeast extract, and 0.5% (w/v) NaCl) supplemented with 15 μg/ml chloramphenicol and 10% glycerol in 96 deep-well plates. The second multi-substrate functional screens were carried out in peptone-free nutrient agar (NA; 0.3% (w/v) yeast extract, 0.5% (w/v) NaCl, 1.5% (w/v) agar) supplemented with 15 μg/ml chloramphenicol and 0.1% (w/v) L-arabinose. A double strength solution of temperate NA containing L-arabinose and antibiotic was mixed with the appropriate volume of a set of substrates prepared as follows. A final concentration of 1% (w/v) skim milk and 0.04% (w/v) cold water fish skin gelatin (Sigma-Aldrich, USA) and 0.5% (w/v) blood meal (Giva, Sweden) were respectively added from a sterile stock solution previously autoclaved at 116 °C for 20 min. An 8% (w/v) zein from maize (Sigma-Aldrich, USA) solution was dissolved in 100% dimethyl sulfoxide (DMSO). Other insoluble substrates such as wheat gluten (Sigma-Aldrich, USA), bone meal (Weibulls, Sweden), and feather meal (Skretting AS, Norway) were directly mixed in the NA solution in parallel. To prevent aggregation of zein, gluten, bone meal, and feather meal when mixed with nutrient agar, the solution was stirred before and during pouring (Wehrle et al. 1999). The NA-substrate solution was topped to 50 ml with pre-warmed (60 °C) sterile water, gently mixed, poured into 12 × 12 cm plates, and left to solidify at room temperature. The plates were air-dried inside the laminar flow cabinet before inoculation to avoid condensation droplets that might result in cross-contamination. Inoculation was done manually with a 96-pin replicator in quadruple. Two negative controls consisting of non-proteolytic EPI300-T1^R^ candidate clones on skim milk were used. Plates were then incubated upside-down for 14–16 h at 37 °C inside a plastic bag to maintain humidity. Development of clearing zone was followed over 2–3 days at room temperature. Temperature screens were assayed with parallel incubations at different temperatures. In this case, two identical plates were inoculated with the collection of proteolytic fosmid clones. Colony growth and clearing zone formation from substrate hydrolysis were registered after an overnight incubation at 37 °C. Subsequently, plates were incubated in parallel for 2–3 more days at room temperature (approximately 20–22 °C) as a gold standard in functional screens and at 55 °C, a typical temperature used during biomass processing, while clearing zone development was monitored. While most of the substrates rendered visible clearing zones in peptone-free medium, feather, bone meal and fish gelatin plates were flooded with Coomassie stain to increase contrast of clearing zones. Plates were then incubated at room temperature during gentle shaking for at least 1 h or until the first 2–3 mm agar were densely stained.

### Sequencing, annotation, protease candidate selection, and taxonomic assignment

The selected clones were grown in 40 ml LB medium supplemented with 15 μg/ml chloramphenicol at 37 °C in the absence of high-copy inducer. Cells were pelleted and fosmid DNA isolated with NucleoSpin Plasmid kit (Macherey-Nagen, Germany) following the protocol for low-copy plasmid propagation. After elution, extracts were treated with Plasmid-Safe ATP-dependent DNase (Epicentre, USA) to eliminate genomic DNA that may interfere in the downstream sequencing. Proteolytic EPI300-T1^R^ clones were subjected to terminal Sanger sequencing at Secugen (Madrid, Spain). Unique fosmids were then sequenced in pools using a MiSeq Sequencing System (Illumina, San Diego, USA) with a 2 × 150-bp sequencing kit. The fosmid metagenomic DNA sequence was submitted to Rapid Annotation using Subsystem Technology (RAST) for gene annotation (Aziz et al. 2008). In parallel, open reading frames (ORFs) were searched by Blast (Altschul et al. 1990) against the MEROPS (Rawlings et al. 2018) and NCBI databases. Sequence identity was noted relative to the closest non-identical sequence. Multiple sequence alignments were built and used to extract positional information of conserved catalytic residues. Pfam (Finn et al. 2014) and CDD (Marchler-Bauer et al. 2011) were used to predict the domain structure. SignalP v4.1 (Nielsen 2017) was used to predict signal peptide sequences. Taxonomic assignment was performed using the MetaErg tool, which classifies all open reading frames in a contig based on best DIAMOND hits against a custom database, GenomeDB (Dong and Strous 2019).

### Recombinant expression of proteases and functional screen

The candidate protease sequences selected from the proteolytic clones were used as templates for gene synthesis, and genes were codon-optimized by GenScript to maximize expression in *E. coli*, with the exception of the C6 candidate*.* Predicted domain structure was used as a guide for truncations. Genes were flanked by *Sap*I restriction sites and delivered in a customized *Sap*I-free pUC57 plasmid with kanamycin selection marker (GenScript, USA). The encoding DNA sequence for candidate C6 was PCR-amplified with Phusion High Fidelity DNA polymerase (NEB, UK) from the MH1 fosmid clone, using the *Sap*I site-containing primers MH1F 5′-atatatgctcttctagtaaaggcgaaaactacgaccacgatccc-3′ and MH1R 5′-tatatagctcttcatgcaacacgtgtctgagtcgcggccgc-3′ (restriction sites underlined). The gene fragments were sub-cloned from the delivery vector, or directly from the PCR amplicon in the case of C6, into two expression vectors, p1 and p12, supporting arabinose-induced expression of N-terminal and C-terminal histidine (his) fusion proteins, respectively, in *E. coli* MC1061 (Bjerga et al. 2016). A version of each expression vector, containing a hexapeptide-coding linker (encoding a GSGSGS peptide), was used as background controls. Construction of the expression vectors as well as expression and partial characterization were done as previously described (Bjerga et al. 2016). Following expression, cells were harvested, resuspended in 1 ml lysis buffer (50 mM Tris HCl pH 8.5, 50 mM NaCl, 0.25 mg/ml lysozyme, 10% (v/v) glycerol), and sonicated as described above. Cleared lysates were stored at − 20 °C and used directly for proteolytic activity assessments. Expression and solubility were assessed by TGX precast SDS-PAGE gels in a Mini PROTEAN electrophoresis system (Bio-Rad, Spain). Gels were stained with SimplyBlue SafeStain (Thermo Scientific, USA). The substrate range activity of the recombinant enzymes was analyzed following a similar approach as for the second fosmid clone screen. Plates with the seven different proteinaceous substrates were prepared as described above but supplemented with 100 μg/ml ampicillin and 0.1% (w/v) L-arabinose. In this case, overnight precultures of both constructs were inoculated in fresh LB medium, grown for 4 h under constant agitation at 37 °C followed by induction with 0.1% (w/v) L-arabinose for 3 more hours. Inoculation to plates was done with a 96-pin replicator, in triplicates. Plates were incubated for 16–20 h at 37 °C for colony development and further overnight at 55 °C for cell lysis and halo development. Coomassie staining was done as described before for the fish gelatin, feather, and bone meal plates.

### Proteolytic activity from cell lysates on zein

A mixture consisting of 9 ml assay buffer (50 mM Tris HCl pH 8.5, 50 mM NaCl, 10% glycerol (v/v)), 1 ml of cell lysate, and 0.3 g zein from maize (Sigma-Aldrich, USA) was prepared in 15 ml polyethylene (PE) round-bottom tubes. The tubes were vortexed in order to resuspend the zein powder and incubated at 20 and 50 °C under constant mixing on a wheel for 48 h. Three replicates per samples were run in parallel. Hydrolysis and solubilization of zein was monitored by detection of TCA-soluble peptides. Standard curve was made with 0–5.5 mM L-tyrosine (Sigma-Aldrich, USA). Soluble L-tyrosine and L-tryptophan containing zein peptides specifically react with Folin-Ciocalteu reagent (Sigma-Aldrich, USA) to produce a color change with a maximum absorbance at 660 nm. The tubes were centrifuged at 12,000×*g* for 10 min in order to pellet insoluble proteins. Samples were taken avoiding the top carotenoid-rich layer and further diluted in order to fit within the standard curve range. Measurements were done in triplicate in a Hidex Sense microplate reader (Hidex, Finland).

### Purification and characterization of the C6 protease

The his-tagged C6 protein was expressed in *E. coli* MC1061. For enzyme production, a single *E. coli* colony, previously grown at 37 °C on solid LB agar medium supplemented with 100 μg/ml ampicillin, was picked and used to inoculate 50 ml of LB medium supplemented with 100 μg/ml ampicillin in a 0.25 L flask, following by cultivation at 37 °C and 200 rpm overnight. Afterwards, 50 ml of this culture was used to inoculate 1 L of LB medium with antibiotic in a 2.5 L flask, which was incubated to an OD_600nm_ of approximately 0.7 at 37 °C. Protein expression was induced by adding L-arabinose to a final concentration of 0.1% (w/v), followed by incubation for 16 h at 16 °C. The cells were harvested by centrifugation at 5000×*g* for 15 min to yield a pellet of 2–3 g (wet weight). The wet cell pellet was frozen at − 80 °C overnight, thawed and resuspended in 15 ml of 50 mM sodium phosphate, pH 8.0, 10 mM imidazole, and 300 mM NaCl. Lysonase Bioprocessing Reagent (Novagen, Germany) was added (4 μl/g wet cells) and incubated for 60 min on ice with rotating mixing. The cell suspension was sonicated for a total of 5 min and centrifuged at 15,000×*g* for 15 min at 4 °C, and the supernatant was retained. The soluble His-tagged protein was purified at 4 °C by affinity chromatography in Ni-NTA His-Bind (Sigma-Aldrich, USA) and elution with 50 mM sodium phosphate pH 8.0, 250 mM imidazole, and 300 mM NaCl. Eluted protein was subjected to ultra-filtration through low-adsorption hydrophilic 10,000 nominal molecular weight limit cut-off regenerated cellulose membranes (Amicon, USA) to concentrate the protein solution. An extensive dialysis of protein solutions against 40 mM 4-(2-hydroxyethyl)-1-piperazineethanesulfonic acid (HEPES) buffer pH 7.0 was performed using Pur-A-LyzerTM Maxi 1200 dialysis kit (Sigma-Aldrich, USA), as follows; 5 ml concentrated protein solution was dialyzed against 2 L buffer during 1 h at room temperature, after which the buffer was changed by other 2 L buffer and maintained 1 h more. Then, the buffer was changed and the dialysis was kept overnight at 4 °C. The dialyzed protein solution was recovered and concentrated as before. Purity was assessed as > 98% using SDS-PAGE analysis in a Mini PROTEAN electrophoresis system (Bio-Rad, Spain). A total of about 4.6 mg total purified recombinant protein was obtained from 1 L culture.

Optimal pH of the C6 candidate was studied in 50 mM Britton-Robinson buffer at 50 °C, as follows. A stock solution of 10% zein (Sigma Aldrich, USA) was prepared in DMSO. To a total of 200 μl of buffer at the desired pH, 10 μl of the zein stock solution and 2 μl of a stock solution of purified recombinant C6 (30 mg/ml) were added. Triplicate reactions and a control without enzyme were maintained for 2 h at 50 °C. Hydrolysis of zein was monitored by detection of L-tyrosine at 492 nm using the Tyrosine Assay Kit (Sigma-Aldrich, USA). For temperature optima determination, 50 mM Britton-Robinson buffer pH 10.0 and a temperature range from 20 to 70 °C were set up. Zein hydrolysis was monitored, in triplicates with control reactions, at the indicated temperatures for 24 h using the L-tyrosine detection assay kit. Measurements were done in a Synergy HT Multi-Mode Microplate Reader (Bio Tek, USA).

## Results

### Identification of proteolytic enzymes in a two-step functional screen

To identify new proteases from uncultured microbes of environmental metagenomes, we screened four DNA libraries (in the host *E. coli*) containing genomic DNA derived from mix culture collections of marine mesophilic and thermophilic bacteria and uncultured microbial communities inhabiting marine water samples and sediments from Milazzo harbor. Libraries were selected, among a total of about 30 existing clone libraries from microbial communities inhabiting 28 geographically distinct environmental sites, based on broad diversity and high enzyme discovery rates (Leis et al. 2015; Popovic et al. 2017; Martínez-Martínez et al. 2018). The libraries were firstly screened on solid medium for their capacity to hydrolyze skim milk (first screen). In total, 11 proteolytic clones were identified in a total of 340,000 clones tested (Table 2). These served as the starting material for the assessment of new functional screens based on other proteinaceous substrates (second multi-substrate screen).

### Parallel multi-substrate screen at elevated temperature confirmed relevant proteolytic clones

We performed several functional agar-based assays using a number of substrates to identify industry-relevant protease candidates with a broad-substrate scope. In addition to skim milk, seven proteinaceous substrates were selected that included fish gelatin, zein, gluten, bone meal, feather meal, and blood meal. Although some of the insoluble substrates (zein, gluten, bone meal, and feather meal) remained visible on the plates as small agglomerates, stirring resulted in a homogeneous dispersion of the particles, allowing detection of hydrolysis.

**Fig. 1:**
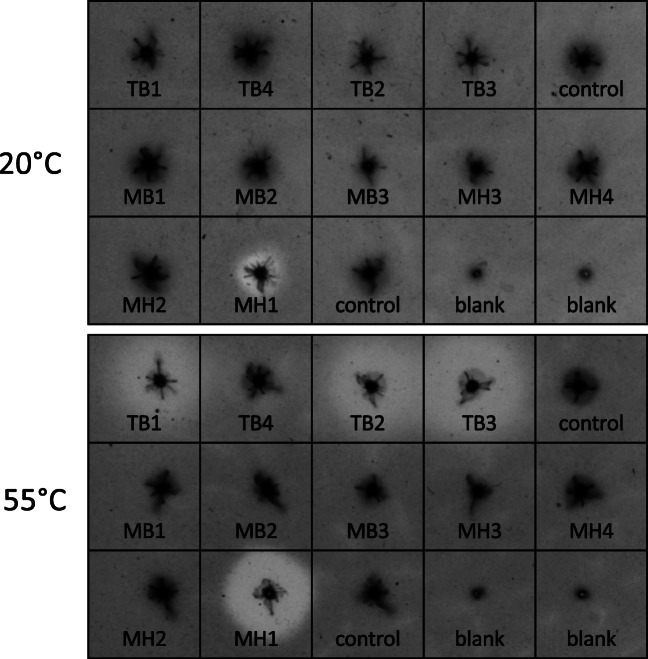
Functional screen of eleven proteolytic EPI300-T1^R^ clones on NA medium supplemented with zein. The plates were grown overnight at 37 °C, and for another 48 h at 20 °C (top) or 55 °C (bottom). The formation of clearing zones around the clones is attributed to enzymatic hydrolysis of the zein substrate. Two different non-proteolytic fosmid clones were used as negative background controls. Two blank spots were included to monitor eventual contamination carried on the pin replicator

The eleven proteolytic fosmid clones that were identified during the first screen step on skim milk were tested for activity against the seven proteinaceous substrates in a second screen step. Two parallel experiments were performed for each substrate by overnight colony growth at 37 °C on solid media followed by up to 72 h incubation at two temperature conditions: one parallel at 20 °C as a standard for functional metagenomic surveys (Table 1) and another parallel at 55 °C, as a common temperature for industrial protein hydrolysis. At the end of the experiment, clearing zone formation, indicating proteolytic activity, was registered and all eleven proteolytic fosmid clones showed reproducible hydrolysis against skim milk (Table 3). Four of the clones, TB1, TB2, TB3, and MH1, showed proteolytic activity against all seven substrates tested. Eight clones showed activity in at least one of the other six substrates, while three clones, MB1, MH3, and MH4, did not show activity against any other substrate. Interestingly the four clones that showed activity against all substrates (MH1, TB1, TB2, and TB3) generated a larger clearing zone after incubation at 55 °C than at 20 °C (Fig. 1). This suggests that they may encode one or more thermophilic/thermostable proteases, which is supported by the fact that these clones originated from metagenomes from mixed mesophilic (MH1) or thermophilic (TB1, TB2, TB3) bacterial strains. Due to their broad-substrate range and potential thermostability, these four clones were selected for sequencing of their metagenomic DNA.

**Table 3:** Summary of the results obtained from the multi-substrate functional screens. Results were monitored after overnight incubation at 37 °C followed by prolonged parallel incubation at 20 and 55 °C temperatures. The overall development of a clearing zone around the colony is taken as an indication of proteolytic activity (plus signs)

Fosmid ID	Clearing zone on agar-based screens							Total
	Skim milk	Fish gelatin	Zein	Gluten	Bone meal	Feather meal	Blood meal	
TB1	+	+	+	+	+	+	+	7
TB2	+	+	+	+	+	+	+	7
TB3	+	+	+	+	+	+	+	7
TB4	+	-	-	+	-	-	+	3
MB1	+	-	-	-	-	-	-	1
MB2	+	-	-	+	-	-	-	2
MB3	+	-	-	+	-	-	-	2
MH1	+	+	+	+	+	+	+	7
MH2	+	+	-	-	-	-	+	3
MH3	+	-	-	-	-	-	-	1
MH4	+	-	-	-	-	-	-	1
Total	11	5	4	7	4	4	6

### Metagenomic DNA of the four clones with broad-substrate range contained 6 putative proteases

**Fig. 2:**
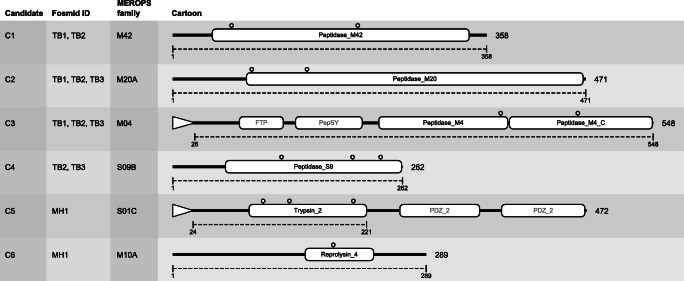
Cartoon of the six proteases candidates with annotated domains. Leader sequences predicted for candidates C1–C6 are indicated by open triangles at the N-termini. Domains and domain names are annotated from Pfam, and depicted as open rounded rectangles. Sequence lengths (amino acids) are indicated to the right of the cartoons. Catalytic residues are extrapolated from multiple sequence alignments of the 20 closest MEROPS homologs and indicated by hairpins. The dotted lines and associated sequence numbers indicate the regions used in recombinant cloning and expression of individual proteases. Cartoon is drawn to scale

Sequencing and annotation of the DNA insert regions revealed that the TB1, TB2, and TB3 clones, which appeared to be active on zein only at 55 °C, contained partially overlapping and identical sequence regions. Their sequences form a single contig (51144 bp length) encoding altogether four candidate protease sequences, two of which are shared among all three clones (Table 2, Table S1). The MH1 clone which appeared to be active on zein at both temperatures tested, but with increased activity at elevated temperatures, contained unique DNA encoding three proteases (Table 2, Table S1). The predicted S54 rhomboid peptidase identified in the clone MH1 was dismissed due to their membrane-associated character and thus lack of industrial compliancy. To conclude, 6 unique putative proteases encoding genes were investigated. The candidates span two serine protease clans (S01, S09) and four metalloproteases clans (M04, M10/12, M20, M42) according to classification by MEROPS, had various length (267–560 amino acids), and their identities to the closest homologous protein sequences ranged from 58.02 to 99.4% (Table 2). Pfam protease domain coordinates and catalytic residues are depicted for all six candidates in Fig. 2. Analysis of the DNA insert region using RAST and MetaErg (data not shown) revealed affiliation of MH1 to *Alphaproteobacteria* and of TB1-TB2-TB3 to *Geobacillus*, in agreement with the microbial diversity of the corresponding samples from which the libraries derived (Leis et al. 2015; Bargiela et al. 2015).

For each of the six gene candidates, constructs were designed for recombinant expression in *E. coli*. The six protease candidates that were subjected to downstream analysis were given an arbitrary identification number, C1–C6 (Table 2). In two of the candidates, predicted leader sequences were excluded. In one candidate, C5, the accessory non-protease PDZ domains were removed to reduce protein size (Fig. 2). Using FX-cloning technology, synthetic gene versions were successfully inserted into two different expression vectors, adding either an N-terminal histidine (his) tag or a C-terminal his-tag, to support recombinant, cytoplasmic expression in *E. coli*, and downstream purification.

#### Expression and activity assessment of recombinant candidates confirmed two soluble proteolytic enzymes

**Fig. 3:**
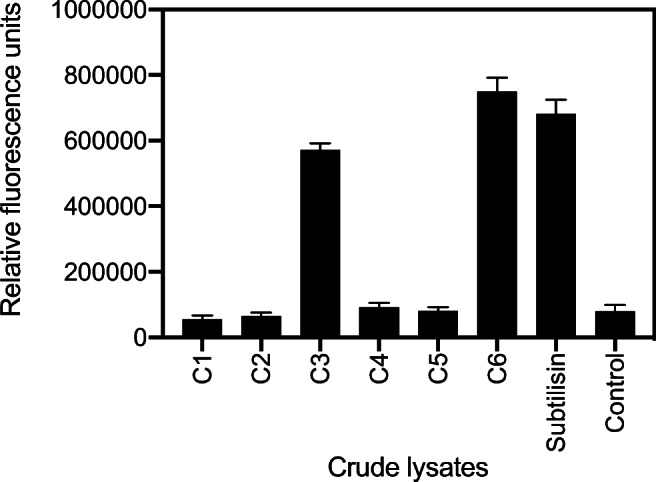
Proteolytic activity of recombinant protease candidates against FITC-casein. Activity in the *E. coli* cell lysates after heterologous expression of C-terminal his-tagged enzymes was assessed after 15 h. A recombinant subtilisin from *B. licheniformis* is shown as positive control (subtilisin). The results are presented as total fluorescence from release of FITC and compared to the background, which was a lysate obtained with a clone encoding a non-proteolytic hexapeptide (GSGSGS) in place of the recombinant protease (control). The values are calculated as the average of three technical replicates of two independent experiments. Error bars indicate standard deviation between all six replicates

Recombinant expression of individual genes (Figure S1) was observed for most of the candidates (except C5), in at least one of the two construct variants, with two of them being unambiguously soluble (C1 and C2). Activity was assessed for all cleared lysates even in the absence of a clear observation of recombinant proteins, based on the assumption and experience that enzymes might be present in small amount, sufficient to identify activity (Bjerga et al. 2016). Two metalloproteases, C3 and C6, revealed proteolytic activity, more than 6-fold above the background control after 15 h incubation on FITC-casein (Fig. 3). A recombinant S8 protease (subtilisin) from *B. licheniformis*, previously used to validate the expression system (Bjerga et al. 2016), showed a similar activity in the experimental conditions. Proteolytic activity was measured for both the N-terminal and C-terminal his-tagged constructs with no significant differences observed (not shown). The four other candidate proteases (C1, C2, C4, and C5) were not shown to be functional, likely due to lack of expression (C5), poor solubility (C4), or no activity of the soluble protein (C1, C2).

Both C3 and C6 are zinc-binding metalloproteases belonging to the MA clan, according to the MEROPS classification. They contain a signature HEXXH sequence, shown by crystallographic studies to form a metal-binding motif, and a glutamate, both important for catalysis (Onoda et al. 2009). The C3 protease was present in TB1, TB2, and TB3 and the C6 protease was present in the MH1 clone. The C3 protease is 548-residue long with a predicted Peptidase_M4 and a Peptidase_M4_C domains according to Pfam (Fig. 2, Table 2). A leader sequence was predicted at the N-terminus. In addition, auxiliary domains FTP and PepSY were predicted at the N-terminal region of the protease (Fig. 2). A MEROPS search identified the closest homolog as a neutral M4 protease with which it shared 98.5% sequence identity (Table 2). The C6 candidate encodes a protease of 289 amino acids with a predicted molecular weight of 32.9 kDa (Fig. 2, Table 2). According to Pfam, the sequence harbors a metalloprotease M12B reprolysin domain across residues 152–229 (Fig. 2). Blast search against the NCBI database identified a homologous metalloprotease sequence which shares a 65% sequence identity (Table 2). A MEROPS search showed that the closest homolog is a M10 protease from *Rhizobium*, which is 58% identical to C6.

#### Both C3 and C6 proteases were confirmed to have a broad-substrate range

**Fig. 4:**
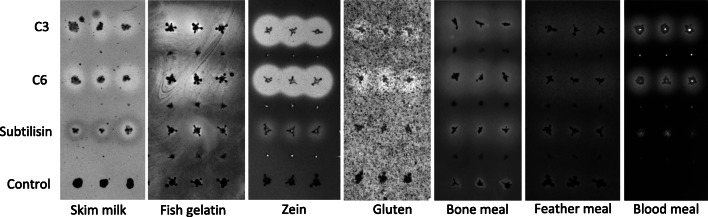
Proteolytic activity of the recombinant protease candidates against the seven proteinaceous substrates. The C3 and C6 candidates were cloned into and expressed recombinantly in *E. coli* with C-terminal his-tags. A recombinantly expressed subtilisin using the same expression system was used as positive control and a non-proteolytic hexapeptide (GSGSGS) in place of the recombinant protease was used as background control. The bacterial cultures were grown and induced in LB before imprinting them in triplicates with a pin replicator onto the NA medium plates supplemented with L-arabinose and ampicillin. The plates were incubated overnight at 37 °C for colony growth, and further for 24–48 h at 55 °C for cell lysis and halo development. Fish gelatin, bone and feather meals plates were Coomassie-stained for improved contrast. The formation of clearing zones around the clones is attributed to enzymatic hydrolysis of the proteinaceous substrate

Both protease candidates were tested for proteolytic activity against the same seven proteinaceous substrates that were used in the multi-substrate screen of the fosmids. A clearing halo was revealed for all substrates (Fig. 4), thus confirming that both C3 and C6 possess a broad-substrate range and suggesting that they are likely responsible for the results obtained during the functional screen.

### One of the proteases was confirmed to completely hydrolyze zein in a liquid assay

**Fig. 5:**
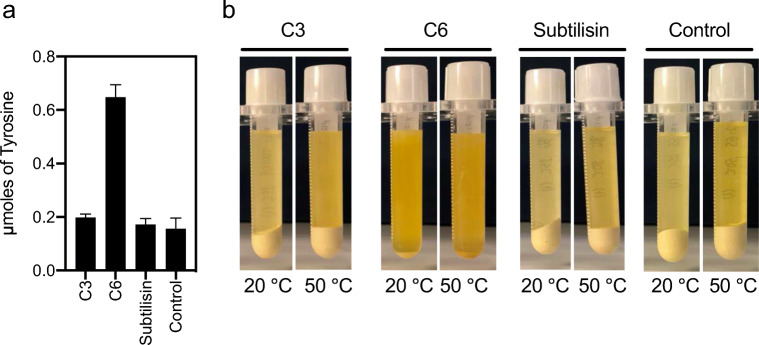
Proteolytic activity of recombinant protease candidates against insoluble zein. **a** Hydrolysis of zein is shown by addition of cleared lysates containing recombinant C3, C6, the *B. licheniformis* subtilisin, and a background obtained with a clone encoding a non-proteolytic hexapeptide (GSGSGS) in place of the recombinant protease (control). The results are presented as μmoles of L-tyrosine available after 48 h hydrolysis of zein based on a standard curve of L-tyrosine and compared to the background. The values are calculated as the average of three technical replicates of two independent experiments. Error bars indicate standard deviation between all six replicates. **b** Solubilization of zein by the C6 protease is clearly visible after a 48 h incubation at 50 °C, as revealed by the reduction of the pellet and the increased opacity of the solution

To assess the zein hydrolyzing capacity of the recombinant C3 and C6 proteases, the cleared *E. coli* lysates obtained from heterologous expression of the protease-encoding genes were produced and incubated with zein at two temperatures, 20 and 50 °C. The hydrolysis result after 48 h incubation (Fig. 5a) was consistent with the observed activities from the screen of the originating fosmid clones on the zein solid agar screens incubated at 55 °C temperature (Table 3, Fig. 1). Almost complete solubilization of zein was observed at 20 and 50 °C for the samples containing C6 compared to background (Fig. 5b). Moreover, C6 already showed significant zein hydrolysis after 24 h incubation at 50 °C and marginal hydrolysis of zein at 20 °C (not shown). The C3 and the recombinant *B. licheniformis* subtilisin rendered only marginal hydrolysis of zein at both temperature conditions tested, comparable to the background. A parallel experiment at pH 7.5 gave the same results (not shown).

**Fig. 6:**
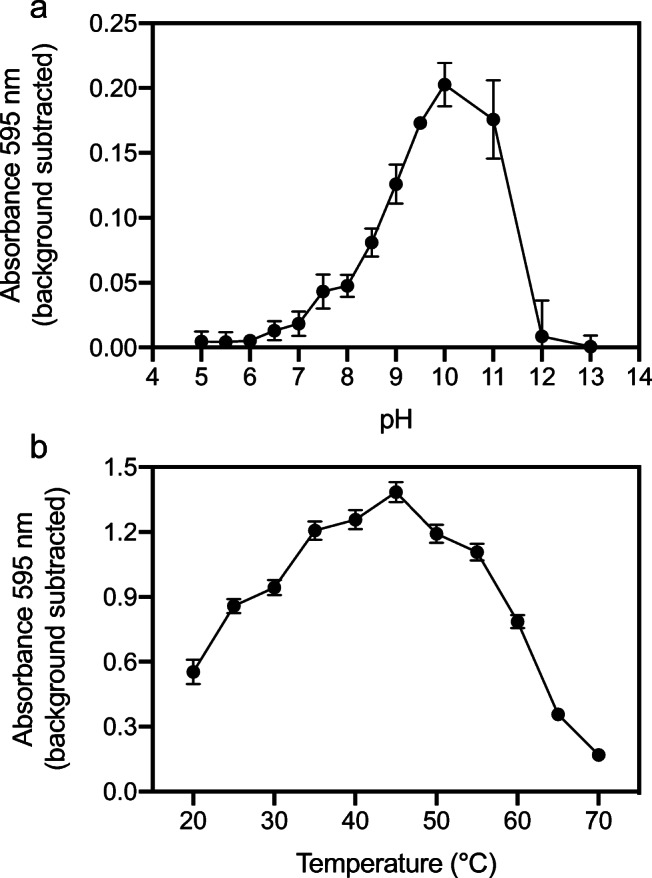
Optimal parameters for activity for the purified C6 protease. Profiles for pH (**a**) and temperature (**b**) were calculated using zein across a pH range from 5.0 to 13.0 (2 h incubation at 50°) and a temperature range from 20 to 70 °C (24 h incubation at pH 10.0). The standard deviation was calculated from three technical replicates

Recombinant C6 protease, purified to 99% purity (Figure S2), displayed highest activity against zein at a range of pH from 9.0 to 11.0, with a maximum at pH 10 (Fig. 6a). Further, C6 protease showed significant hydrolyzing activity against zein at all temperatures tested (20–70 °C) with a maximum observed at 45 °C (Fig. 6b).

## Discussion

Reports on new proteases identified by functional metagenomics from the last two decades encompass a limited substrate range and mild screening conditions (Table 1 and references therein). Altogether, these studies have reported 23 proteases, with a clear bias toward serine proteases, and most ignoring desirable attributes for industrial application. This landscape supports the notion that current standard screening techniques do not suffice for accessing the full repertoire of microbial proteases with potential for industrial applications (Morris and Marchesi 2015). In line with other similar setups for other enzyme classes (Schückel et al. 2016; Maruthamuthu et al. 2016), our study demonstrates the successful use of a two-step screen, and we show that other substrates and conditions for activity assessment may be useful to retrieve new enzymes with broad-substrate range and moderately high temperature stability. Of the eleven proteolytic clones that presented clearing zones in the first screen on SMA plates (Table 3), several did not show hydrolysis in any of the other substrates in the second screen. This discrepancies may indicate that these clones contain hydrolases that are active on other constituents than casein in skim milk, as it has previously been described (Jones et al. 2007). Despite its limitations, we validated skim milk/casein as an easily accessible, soluble and useful substrate for an initial screen. A second screening step of multiple substrates may thus be used to filter out non-proteolytic candidates or proteases with a narrow substrate range. Moreover, the six candidate proteases identified with broadest substrate range belong to two different clans and none of them is related to the frequently found S08 subtilisin type (Table 1, Table 2). The approach presented here may assist to focus the enzyme discovery effort and efficiently eliminate resource-demanding examination of enzymes with limited value for downstream research and applications.

All the mixed-genomic and metagenomic libraries screened originate from marine environments (Table 2). However, none of the proteinaceous substrates chosen, except for cold water fish gelatin, are in principle relevant for the marine environment. This approach is therefore able to discover new enzymes independently of the metagenomic origin toward the activity that is sought. Thus, our study shows the potential of marine bioprospecting to identify new unexplored sequences, in line with previous reports (Ferrer et al. 2019).

Most of the proteinaceous substrates used in this study are insoluble and unable to enter the bacterial *E. coli* cell. The generation of hydrolytic clearing zones relies therefore on the spontaneous lysis of cells to release the enzyme (Tasse et al. 2010; Cárcel-Márquez et al. 2019) or the ability of the surrogate host to export the enzymes coded in the metagenomic DNA. In our approach, an initial incubation at the optimal growth temperature of the host (37 °C) allows colony formation and expression from the metagenomic DNA using *E. coli*’s transcription/translation machinery. Subsequent incubation at lower temperature (20 °C) allows clearing zone formation to proceed, while growth is stalled or slowed down. This strategy is typically used in functional metagenomic surveys for proteases (Table 1) to minimize masking effects due to overgrowth and facilitates the perception of hydrolytic activity (Molitor et al. 2020). However, prolonged parallel incubation at 55 °C accelerates the lysis of cells within the colony and the release of their contents to the medium, thus, increasing the sensitivity of the assay by bypassing secretion routes. This may be particularly true for the MH1 clone, carrying the C6 protease, for which no signal peptide was predicted (Fig. 2) and that repeatedly showed enlarged hydrolytic clearing zones after the prolonged 55 °C incubation step on the substrates it acted on (Fig. 1, Table 3). Increased incubation temperature might also have improved both the solubility of the proteinaceous substrate and the diffusion of the protease.

The method served to identify robust proteases with broad-substrate range, including two proteases (C3 and C6) confirmed to hydrolyze all seven proteinaceous substrates tested in the solid media (Fig. 4).

Zein is of particular interest, as only a limited number of zein-degrading proteases have been reported, all originating from *Bacillus* strains (*B. licheniformis*, *thermoproteolyticus*, *amyloliquefaciens*, *subtilis*, and *pumilus*), the fungus *Aspergillus oryzae* and plants (de Barros and Larkins 1990; Belles et al. 2000; Ramakrishna and Rao 2005; Miyaji et al. 2006). However, the zein-degrading C6 protease (both in solid and liquid assay formats) here reported originate from a bacterium largely neglected for their capacity to degrade zein. Indeed, the insert DNA of the fosmid clone MH1, containing C6 protease, is affiliated to Rhodobacteraceae, a common dweller of marine sediments (Pohlner et al. 2019). The DNA in fosmid clone MH1 originates from a shallow polluted marine sediment and it is likely represented by psychrophiles and mesophiles (Yakimov et al. 2005; Bargiela et al. 2015). Native enzymes from mesophiles can still show temperature tolerance (Veith et al. 2012), which appears to be the case for the C6 protease (Fig. 6). A presumptive thermal-tolerance was also noticed for the C3 protease in solid medium screens. The C3-carrying fosmid clones originate from a collection of thermophilic isolates (Hussein et al. 2015), and the insert DNA sequence affiliated to the *Geobacillus* genus, a known thermophile. Although the C3 protease has a predicted signal peptide for secretion through the ubiquitous Sec pathway (Fig. 1, Fig. 2), hydrolytic activity for the C3-carrying fosmid clones is likely due to cell lysis, as clearing zones appear after prolonged incubations at 37 °C (initial screen on skim milk) and 55 °C (all substrates). Although further biochemical characterization of the C3 protease will be needed to know its exact temperature range of activity, it appears in our study to be moderately thermostable in the solid medium screens. To conclude, this study reports proteases with activity against zein identified with functional metagenomics and demonstrates the power of using multiple proteinaceous substrates to focus efforts in functional metagenomics. In addition to that, adding various temperatures to the two-step solid substrate screen method herein reported may thus, reveal the temperature tolerance of enzymes degrading protein-based substrates.

Although phylogenetic, structural and mechanistic characterization of the C6 protease will be needed to determine its functional novelty; the observed hydrolysis and solubilization of zein at 50–55 °C (Fig. 1, Fig. 5) supports its applicability and robustness in industrial processes. Its alkaline pH profile (Fig. 6b) is distinct from those of previously reported neutral zein-hydrolytic proteases (de Barros and Larkins 1990; Belles et al. 2000; Murakami and Hirata 2000; Ramakrishna and Rao 2005; Miyaji et al. 2006). Subtilisins, and more recently, thermolysins, have been typically used in zein hydrolysis (Murakami and Hirata 2000; Miyaji et al. 2006), based on their commercial availability, but with limited success. In contrast to previous reports using Alcalase® (alkaline subtilisin from *B. licheniformis*) and Neutrase® (neutral thermolysin from *B. amyloliquefaciens*) (Liang et al. 2018), neither the *B. licheniformis* subtilisin nor the thermolysin-like C3 protease rendered activity in our batch zein hydrolysis experiments (Fig. 5). Although we establish that all three enzymes were capable of hydrolyzing casein over 15 h (Fig. 3), the reason why subtilisin and C3 fail in hydrolyzing zein may be explained by our relatively low load of recombinant enzyme to substrate compared to previous reports using commercial enzymes. Although no activity was detected with the other cloned proteases from the TB metagenomic fosmid clones, a cumulative effect during the hydrolysis of zein in the secondary screen step cannot be ruled out. The C6 protease, however, complements the existing catalog of zein-degrading enzymes and may deserve a more exhaustive analysis in industrial zein hydrolysis.

The resolution of classical functional screening strategies for proteases from environmental metagenomic libraries can be increased. The inclusion of a secondary screen with several targeted proteinaceous substrates and industry mimicking conditions can extend its potential for the discovery of new enzymes with industrial relevance. The two-step multi-substrate approach herein described led to distinctive discrimination of enzymes with broad-substrate scopes, including the identification of two thermostable proteases, whereof one readily solubilized zein. This is the first metagenome-derived protease with proven hydrolyzing activity against the insoluble zein, also from a microorganism whose ability to degrade zein was unknown. Altogether, the new functional screening platform can be beneficial to direct future bioprospecting campaigns of proteases toward specific industry applications, thus limiting costly and time-consuming experimental validation to an exclusive set of candidates.

## Supplementary Information

ESM 1 (PDF 4398 kb)

## Data Availability

Metagenomic fosmid DNA sequences have been deposited in GenBank under BioSample accession numbers: SAMN14533297 (MH1) and SAMN14533298 (TB2).
