# Supplementary material for: Two-step functional screen on multiple proteinaceous substrates reveals temperature-robust proteases with a broad-substrate range
Source: Appl Microbiol Biotechnol. 2021 Mar 26;105(8):3195–209. doi: 10.1007/s00253-021-11235-9 (PMC8053189; doi:10.1007/s00253-021-11235-9)

Two-step functional screen on multiple proteinaceous substrates reveals temperature-robust proteases with a broad substrate range.

Applied Microbiology and Biotechnology

Antonio García-Moyano (ORCID 0000-0003-1997-8589)[1], Yuleima Diaz[1], José Navarro[2], David Almendral[2], Pål Puntervoll (0000-0003-0093-7816)[1] , Manuel Ferrer (ORCID 0000-0003-4962-4714)[2] and Gro Elin Kjæreng Bjerga (ORCID 0000-0001-5152-5139)[1]

[1] NORCE Norwegian Research Centre, Thormøhlens gate 55, 5006 Bergen, Norway

[2] Institute of Catalysis, Consejo Superior de Investigaciones Científicas, 28049 Madrid, Spain

Corresponding Author:

Gro Elin Kjæreng Bjerga grbj@norceresearch.no

+47-56 10 74 04

Nygårdsgaten 12, 5008 Bergen, Norway

Table S1 ORF prediction and annotation by RASTtk for the metagenomic DNA in the four proteolytic clones TB1, TB2, TB3 and MH1

Predicted protease ORFs are marked in bold. Note that the C6 candidate (zeinase) was only identified as a hypothetical protein-encoding gene by the RAST pipeline. Further manual inspection against the MEROPS database predicted a metalloprotease related sequence.

Clone	Feature ID	Start	Stop	Length (bp)	Function	Subsystems
	fig|6666666.594338.peg.1	1001	9	993	Catabolite control protein A	- none -
	fig|6666666.594338.peg.2	2465	1383	1083	Chorismate mutase I (EC 5.4.99.5) / 2-keto-3-deoxy-D-arabino-heptulosonate-7-phosphate synthase I beta (EC 2.5.1.54)	Phenylalanine and Tyrosine Branches from Chorismate
	fig|6666666.594338.peg.3	3020	2694	327	Uncharacterized protein YtxJ	- none -
	fig|6666666.594338.peg.4	3429	3034	396	Uncharacterized protein YtxH	- none -
	fig|6666666.594338.peg.5	3865	3416	450	UPF0478 protein YtxG	- none -
	fig|6666666.594338.peg.6	5317	4013	1305	UDP-N-acetylmuramate--L-alanine ligase (EC 6.3.2.8)	- none -
	fig|6666666.594338.peg.7	7724	5445	2280	DNA translocase FtsK	- none -
	fig|6666666.594338.peg.8	8386	7781	606	Phenylalanyl-tRNA synthetase domain protein (Bsu YtpR)	- none -
TB1	fig|6666666.594338.peg.9	9205	8408	798	FIG002434: Uncharacterized protein YtpQ	- none -
	fig|6666666.594338.peg.10	9785	9252	534	Inosine/xanthosine triphosphatase	- none -
	**fig|6666666.594338.peg.11**	**10943**	**9867**	**1077**	**Glutamyl aminopeptidase (EC 3.4.11.7); Deblocking aminopeptidase**	**- none -**
	fig|6666666.594338.peg.12	11049	11345	297	Uncharacterized protein YtzB	- none -
	fig|6666666.594338.peg.13	12197	11340	858	Quorum-quenching lactonase YtnP	- none -
	fig|6666666.594338.peg.14	13307	12660	648	tRNA (guanine(46)-N(7))-methyltransferase (EC 2.1.1.33)	- none -
	fig|6666666.594338.peg.15	13447	13716	270	Uncharacterized protein YtzH	- none -
	fig|6666666.594338.peg.16	14554	13769	786	Putative phosphotransferase YtmP	- none -
	fig|6666666.594338.peg.17	16933	14753	2181	Pullulanase (EC 3.2.1.41)	- none -
	fig|6666666.594338.peg.18	17797	17039	759	hypothetical protein	- none -

	fig|6666666.594338.peg.19	18007	17873	135	hypothetical protein	- none -
	fig|6666666.594338.peg.20	18557	17979	579	2'-5' RNA ligase	RNA processing orphans
	fig|6666666.594338.peg.21	19738	18554	1185	Probable 3-phenylpropionic acid transporter	- none -
	**fig|6666666.594338.peg.22**	**21222**	**19807**	**1416**	**Putative dipeptidase YtjP**	**- none -**
	fig|6666666.594338.peg.23	21378	21262	117	hypothetical protein	- none -
	fig|6666666.594338.peg.24	21494	21694	201	Transcriptional regulator, DeoR family	- none -
	fig|6666666.594338.peg.25	22536	21790	747	SSU rRNA pseudouridine(516) synthase (EC 5.4.99.19)	- none -
	fig|6666666.594338.peg.26	22641	22853	213	hypothetical protein	- none -
	fig|6666666.594338.peg.27	24826	23198	1629	Probable cell division protein YtgP	- none -
	fig|6666666.594338.peg.28	24933	26249	1317	Uncharacterized flavin-containing protein YtfP	- none -
	fig|6666666.594338.peg.29	26246	26437	192	hypothetical protein	- none -
	fig|6666666.594338.peg.30	26654	26442	213	Sporulation protein cse60	- none -
	**fig|6666666.594338.peg.31**	**28383**	**26737**	**1647**	**Thermolysin (EC 3.4.24.27)**	**- none -**
	fig|6666666.594338.peg.32	28777	28424	354	hypothetical protein	- none -
	fig|6666666.594338.peg.33	28936	28814	123	hypothetical protein	- none -
	fig|6666666.594338.peg.34	29367	29053	315	Rhodanese-like domain protein	- none -
	fig|6666666.594338.peg.35	32024	29607	2418	Leucyl-tRNA synthetase (EC 6.1.1.4)	tRNA aminoacylation, Leu
	fig|6666666.594338.peg.36	33584	32385	1200	Uncharacterized MFS-type transporter YttB	- none -
	fig|6666666.594338.peg.37	33864	33700	165	hypothetical protein	- none -
	fig|6666666.594338.peg.38	34260	33997	264	Uncharacterized protein YtzC	- none -
	fig|6666666.594338.peg.39	34412	34729	318	[4Fe-4S]-AdoMet protein YtqA	- none -
	fig|6666666.594338.peg.40	34726	35304	579	Putative rRNA methylase YtqB	- none -
	fig|6666666.594338.peg.41	36444	35362	1083	Tetraprenyl-beta-curcumene synthase (EC 4.2.3.130)	- none -
	fig|6666666.594338.peg.42	36645	36484	162	hypothetical protein	- none -
	fig|6666666.594338.peg.43	36664	37206	543	Carbonic anhydrase, gamma class (EC 4.2.1.1)	- none -
	fig|6666666.594338.peg.44	38486	37275	1212	S-adenosylmethionine synthetase (EC 2.5.1.6)	- none -
	fig|6666666.594338.peg.45	38902	40494	1593	Phosphoenolpyruvate carboxykinase [ATP] (EC 4.1.1.49)	- none -
	fig|6666666.594338.peg.46	41567	40686	882	hypothetical protein	- none -
TB2	fig|6666666.594339.peg.1	2089	2	2088	DNA translocase FtsK	- none -
	fig|6666666.594339.peg.2	2778	2173	606	Phenylalanyl-tRNA synthetase domain protein (Bsu YtpR)	- none -
	fig|6666666.594339.peg.3	3597	2800	798	FIG002434: Uncharacterized protein YtpQ	- none -

fig|6666666.594339.peg.4	4174	3644	531	Inosine/xanthosine triphosphatase	- none -
**fig|6666666.594339.peg.5**	**5335**	**4259**	**1077**	**Glutamyl aminopeptidase (EC 3.4.11.7); Deblocking aminopeptidase**	**- none -**
fig|6666666.594339.peg.6	5441	5737	297	Uncharacterized protein YtzB	- none -
fig|6666666.594339.peg.7	6589	5732	858	Quorum-quenching lactonase YtnP	- none -
fig|6666666.594339.peg.8	6738	6619	120	hypothetical protein	- none -
fig|6666666.594339.peg.9	7699	7052	648	tRNA (guanine(46)-N(7))-methyltransferase (EC 2.1.1.33)	- none -
fig|6666666.594339.peg.10	7840	7727	114	hypothetical protein	- none -
fig|6666666.594339.peg.11	7839	8108	270	Uncharacterized protein YtzH	- none -
fig|6666666.594339.peg.12	8946	8161	786	Putative phosphotransferase YtmP	- none -
fig|6666666.594339.peg.13	11325	9145	2181	Pullulanase (EC 3.2.1.41)	- none -
fig|6666666.594339.peg.14	12366	11431	936	hypothetical protein	- none -
fig|6666666.594339.peg.15	12949	12371	579	2'-5' RNA ligase	RNA processing orphans
fig|6666666.594339.peg.16	14133	12955	1179	Probable 3-phenylpropionic acid transporter	- none -
**fig|6666666.594339.peg.17**	**15616**	**14204**	**1413**	**Putative dipeptidase YtjP**	**- none -**
fig|6666666.594339.peg.18	15862	16083	222	Transcriptional regulator, DeoR family	- none -
fig|6666666.594339.peg.19	16929	16183	747	SSU rRNA pseudouridine(516) synthase (EC 5.4.99.19)	- none -
fig|6666666.594339.peg.20	17034	17246	213	hypothetical protein	- none -
fig|6666666.594339.peg.21	19088	17463	1626	Probable cell division protein YtgP	- none -
fig|6666666.594339.peg.22	19195	20511	1317	Uncharacterized flavin-containing protein YtfP	- none -
fig|6666666.594339.peg.23	20523	20699	177	hypothetical protein	- none -
fig|6666666.594339.peg.24	20841	20704	138	Sporulation protein cse60	- none -
fig|6666666.594339.peg.25	20950	20822	129	Sporulation protein cse60	- none -
**fig|6666666.594339.peg.26**	**22679**	**21033**	**1647**	**Thermolysin (EC 3.4.24.27)**	**- none -**
fig|6666666.594339.peg.27	22705	22875	171	hypothetical protein	- none -
fig|6666666.594339.peg.28	23232	23110	123	hypothetical protein	- none -
fig|6666666.594339.peg.29	23663	23349	315	Rhodanese-like domain protein	- none -
fig|6666666.594339.peg.30	26320	23903	2418	Leucyl-tRNA synthetase (EC 6.1.1.4)	tRNA aminoacylation, Leu
fig|6666666.594339.peg.31	27880	26681	1200	Uncharacterized MFS-type transporter YttB	- none -
fig|6666666.594339.peg.32	28160	27996	165	hypothetical protein	- none -
fig|6666666.594339.peg.33	28574	28293	282	Uncharacterized protein YtzC	- none -

	fig|6666666.594339.peg.34	28708	29025	318	[4Fe-4S]-AdoMet protein YtqA	- none -
	fig|6666666.594339.peg.35	29013	29600	588	Putative rRNA methylase YtqB	- none -
	fig|6666666.594339.peg.36	30740	29658	1083	Tetraprenyl-beta-curcumene synthase (EC 4.2.3.130)	- none -
	fig|6666666.594339.peg.37	30941	30780	162	hypothetical protein	- none -
	fig|6666666.594339.peg.38	30960	31502	543	Carbonic anhydrase, gamma class (EC 4.2.1.1)	- none -
	fig|6666666.594339.peg.39	32782	31571	1212	S-adenosylmethionine synthetase (EC 2.5.1.6)	- none -
	fig|6666666.594339.peg.40	33198	34790	1593	Phosphoenolpyruvate carboxykinase [ATP] (EC 4.1.1.49)	- none -
	fig|6666666.594339.peg.41	35959	34982	978	hypothetical protein	- none -
	fig|6666666.594339.peg.42	36320	36204	117	hypothetical protein	- none -
	fig|6666666.594339.peg.43	36462	36671	210	hypothetical protein	- none -
	fig|6666666.594339.peg.44	36676	37923	1248	Uncharacterized MFS-type transporter	- none -
	fig|6666666.594339.peg.45	37937	38092	156	hypothetical protein	- none -
	fig|6666666.594339.peg.46	39146	38196	951	hypothetical protein	- none -
	fig|6666666.594339.peg.47	39303	39172	132	hypothetical protein	- none -
	fig|6666666.594339.peg.48	39622	39380	243	Uncharacterized protein YtmB	- none -
	**fig|6666666.594339.peg.49**	**40479**	**39691**	**789**	**Dipeptidyl aminopeptidases/acylaminoacyl-peptidase**	**- none -**
	fig|6666666.594339.peg.50	40772	41161	390	ABC transporter, substrate-binding protein (cluster 10, nitrate/sulfonate/bicarbonate)	- none -
	fig|6666666.594339.peg.51	41407	41228	180	Phage protein	- none -
	fig|6666666.594339.peg.52	41971	41420	552	Phage protein	- none -
	fig|6666666.594339.peg.53	43130	41964	1167	DNA translocase FtsK	- none -
	fig|6666666.594339.peg.54	43425	43255	171	hypothetical protein	- none -
	fig|6666666.594339.peg.55	44666	43866	801	Phage protein	- none -
	fig|6666666.594339.peg.56	45394	44768	627	N-acetylmuramoyl-L-alanine amidase (EC 3.5.1.28)	Murein Hydrolases, Recycling of Peptidoglycan Amino Acids
TB3	fig|6666666.594340.peg.1	2296	116	2181	Pullulanase (EC 3.2.1.41)	- none -
	fig|6666666.594340.peg.2	3337	2402	936	hypothetical protein	- none -
	fig|6666666.594340.peg.3	3920	3342	579	2'-5' RNA ligase	RNA processing orphans
	fig|6666666.594340.peg.4	5104	3926	1179	Probable 3-phenylpropionic acid transporter	- none -
	**fig|6666666.594340.peg.5**	**6587**	**5175**	**1413**	**Putative dipeptidase YtjP**	**- none -**
	fig|6666666.594340.peg.6	6743	7054	312	Transcriptional regulator, DeoR family	- none -
	fig|6666666.594340.peg.7	7900	7154	747	SSU rRNA pseudouridine(516) synthase (EC 5.4.99.19)	- none -

fig|6666666.594340.peg.8	8471	8049	423	hypothetical protein	- none -
fig|6666666.594340.peg.9	10186	8558	1629	Probable cell division protein YtgP	- none -
fig|6666666.594340.peg.10	10299	11609	1311	Uncharacterized flavin-containing protein YtfP	- none -
fig|6666666.594340.peg.11	11621	11797	177	hypothetical protein	- none -
fig|6666666.594340.peg.12	12014	11802	213	Sporulation protein cse60	- none -
**fig|6666666.594340.peg.13**	**13743**	**12097**	**1647**	**Thermolysin (EC 3.4.24.27)**	**- none -**
fig|6666666.594340.peg.14	14137	13784	354	hypothetical protein	- none -
fig|6666666.594340.peg.15	14296	14174	123	hypothetical protein	- none -
fig|6666666.594340.peg.16	14727	14413	315	Rhodanese-like domain protein	- none -
fig|6666666.594340.peg.17	17384	14967	2418	Leucyl-tRNA synthetase (EC 6.1.1.4)	tRNA aminoacylation, Leu
fig|6666666.594340.peg.18	18944	17745	1200	Uncharacterized MFS-type transporter YttB	- none -
fig|6666666.594340.peg.19	19224	19060	165	hypothetical protein	- none -
fig|6666666.594340.peg.20	19620	19357	264	Uncharacterized protein YtzC	- none -
fig|6666666.594340.peg.21	19772	20089	318	[4Fe-4S]-AdoMet protein YtqA	- none -
fig|6666666.594340.peg.22	20077	20664	588	Putative rRNA methylase YtqB	- none -
fig|6666666.594340.peg.23	21804	20722	1083	Tetraprenyl-beta-curcumene synthase (EC 4.2.3.130)	- none -
fig|6666666.594340.peg.24	22005	21844	162	hypothetical protein	- none -
fig|6666666.594340.peg.25	22024	22566	543	Carbonic anhydrase, gamma class (EC 4.2.1.1)	- none -
fig|6666666.594340.peg.26	23855	22635	1221	S-adenosylmethionine synthetase (EC 2.5.1.6)	- none -
fig|6666666.594340.peg.27	24262	25854	1593	Phosphoenolpyruvate carboxykinase [ATP] (EC 4.1.1.49)	- none -
fig|6666666.594340.peg.28	27023	26046	978	hypothetical protein	- none -
fig|6666666.594340.peg.29	27384	27268	117	hypothetical protein	- none -
fig|6666666.594340.peg.30	27686	28987	1302	Uncharacterized MFS-type transporter	- none -
fig|6666666.594340.peg.31	29001	29156	156	hypothetical protein	- none -
fig|6666666.594340.peg.32	30210	29260	951	hypothetical protein	- none -
fig|6666666.594340.peg.33	30367	30236	132	hypothetical protein	- none -
fig|6666666.594340.peg.34	30686	30444	243	Uncharacterized protein YtmB	- none -
**fig|6666666.594340.peg.35**	**31543**	**30755**	**789**	**Dipeptidyl aminopeptidases/acylaminoacyl-peptidase**	**- none -**
fig|6666666.594340.peg.36	31836	32225	390	ABC transporter, substrate-binding protein (cluster 10, nitrate/sulfonate/bicarbonate)	- none -
fig|6666666.594340.peg.37	32471	32292	180	Phage protein	- none -
fig|6666666.594340.peg.38	33035	32484	552	Phage protein	- none -

	fig|6666666.594340.peg.39	34194	33028	1167	DNA translocase FtsK	- none -
	fig|6666666.594340.peg.40	34489	34319	171	hypothetical protein	- none -
	fig|6666666.594340.peg.41	35730	34930	801	Phage protein	- none -
	fig|6666666.594340.peg.42	36458	35832	627	N-acetylmuramoyl-L-alanine amidase (EC 3.5.1.28)	Murein Hydrolases, Recycling of Peptidoglycan Amino Acids
MH1	fig|6666666.594341.peg.1	844	2	843	Trk potassium uptake system protein TrkH	- none -
	fig|6666666.594341.peg.2	1131	892	240	hypothetical protein	- none -
	fig|6666666.594341.peg.3	1867	1406	462	2-hydroxychromene-2-carboxylate isomerase	- none -
	fig|6666666.594341.peg.4	3522	2419	1104	GTP cyclohydrolase I (EC 3.5.4.16) type 2	- none -
	fig|6666666.594341.peg.5	4849	3665	1185	O-acetylhomoserine sulfhydrylase (EC 2.5.1.49)	- none -
	fig|6666666.594341.peg.6	5113	6039	927	hypothetical protein	- none -
	fig|6666666.594341.peg.7	6069	6758	690	Glutathione S-transferase (EC 2.5.1.18)	- none -
	fig|6666666.594341.peg.8	7035	6838	198	Mobile element protein	- none -
	fig|6666666.594341.peg.9	7907	7050	858	hypothetical protein	- none -
	**fig|6666666.594341.peg.10**	**8041**	**8778**	**738**	**Rhomboid family protein**	**- none -**
	fig|6666666.594341.peg.11	9626	8841	786	Inositol-1-monophosphatase (EC 3.1.3.25)	- none -
	fig|6666666.594341.peg.12	9733	10758	1026	Putative oxidoreductase YncB	Broadly distributed proteins not in subsystems, Quinone oxidoreductase family
	fig|6666666.594341.peg.13	12242	11298	945	Transcriptional activator MetR	LysR-family proteins in Escherichia coli, LysR-family proteins in Salmonella enterica Typhimurium
	fig|6666666.594341.peg.14	12420	13283	864	5,10-methylenetetrahydrofolate reductase (EC 1.5.1.20)	One-carbon metabolism by tetrahydropterines
	fig|6666666.594341.peg.15	13344	13826	483	Thioesterase family protein	- none -
	fig|6666666.594341.peg.16	14949	13867	1083	peptidoglycan binding domain protein	- none -
	fig|6666666.594341.peg.17	17224	14927	2298	Xanthine dehydrogenase family protein, large subunit	- none -
	**fig|6666666.594341.peg.18**	**18847**	**17378**	**1470**	**HtrA protease/chaperone protein**	**Periplasmic Stress Response**
	fig|6666666.594341.peg.19	20136	19072	1065	hypothetical protein	- none -
	fig|6666666.594341.peg.20	20588	20385	204	hypothetical protein	- none -
	fig|6666666.594341.peg.21	21602	20718	885	HflC protein	Hfl operon
	fig|6666666.594341.peg.22	22759	21602	1158	HflK protein	Hfl operon
	fig|6666666.594341.peg.23	24304	22829	1476	Glutathione reductase (EC 1.8.1.7)	Glutathione: Redox cycle
	fig|6666666.594341.peg.24	25107	24319	789	Ribose-5-phosphate isomerase A (EC 5.3.1.6)	- none -

fig|6666666.594341.peg.25	25338	26036	699	hypothetical protein	- none -
fig|6666666.594341.peg.26	26647	26027	621	Intracellular septation protein IspA	- none -
fig|6666666.594341.peg.27	27552	26650	903	Membrane protein	- none -
fig|6666666.594341.peg.28	28621	27608	1014	Signal recognition particle receptor FtsY	- none -
fig|6666666.594341.peg.29	28667	29152	486	hypothetical protein	- none -
**fig|6666666.594341.peg.30**	**29254**	**30123**	**870**	**hypothetical protein**	**- none -**
fig|6666666.594341.peg.31	31868	30174	1695	Tungsten-containing formate dehydrogenase beta subunit	- none -
fig|6666666.594341.peg.32	32167	33237	1071	hypothetical protein	- none -
fig|6666666.594341.peg.33	33224	33556	333	hypothetical protein	- none -
fig|6666666.594341.peg.34	33839	33693	147	hypothetical protein	- none -

Fig. S1 SDS-PAGE analysis of the protease candidates

Recombinant expression of protease candidates (C1 to C6) from two different vectors (p1: N-terminal his-tag; p12: C-terminal his-tag) in E. coli.

Both total (T) and soluble (S) fractions were analysed by SDS-PAGE and stained. Theoretical expected mass is shown under each line.

Overexpressed protein bands are indicated with asterisks.

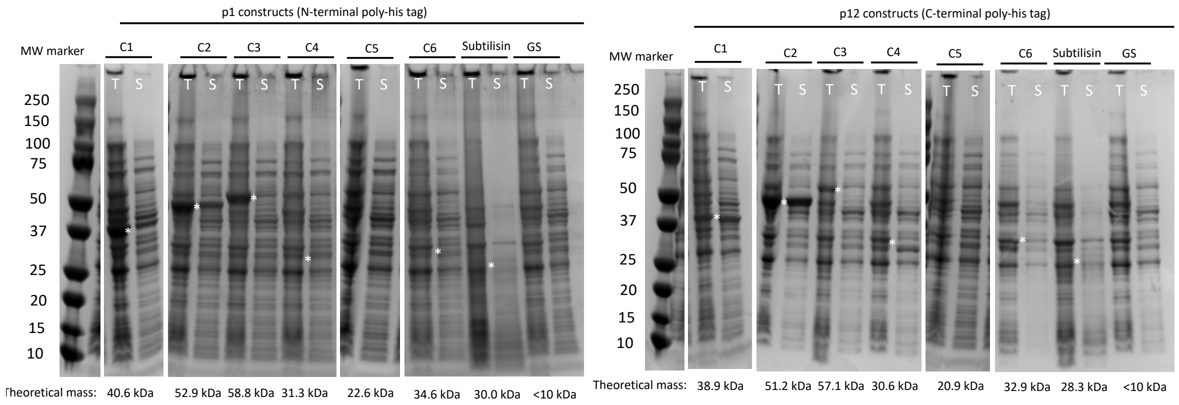

Fig. S2 Protein purity of C6 as determined by SDS-PAGE

Protoblue Safe (National Diagnostics, US) stained SDS-PAGE gel in which a total of 20 µg of protein purified after the C-terminal His_6-tag purification step, is shown. Purity of the protein was high (>99% by densitometry). MW, molecular weight marker; lane 1: cell lysed protein extract containing His_6-tagged C6 protein; lanes 2-6: flow through after binding to resin followed by 5 consecutive washing steps; lane 7, eluted protein.

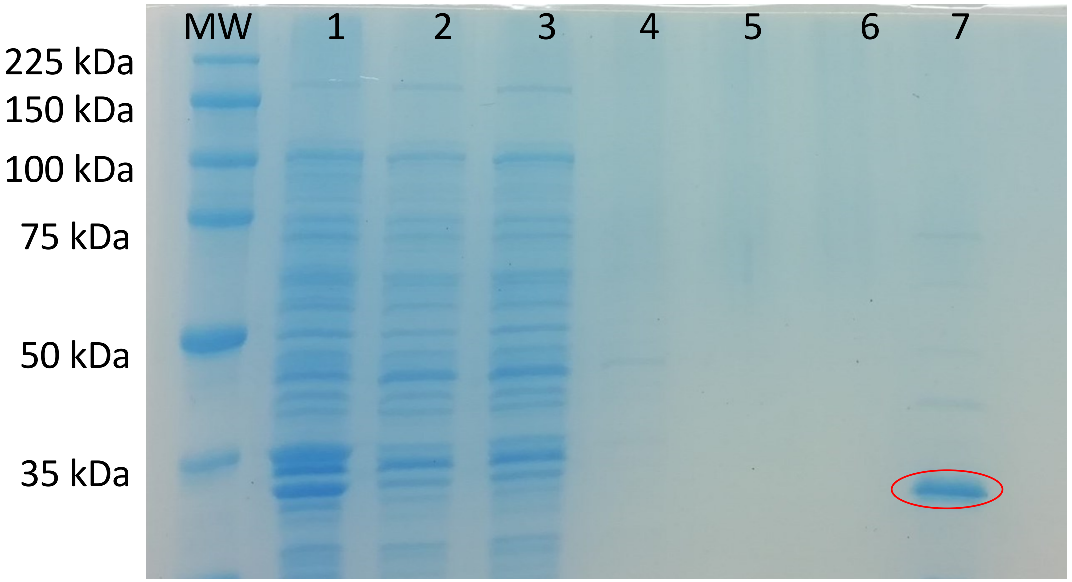

Supplement: Supplementary file 1 — (PDF 4398 kb) [file 253_2021_11235_MOESM1_ESM.pdf]
